# Characterization of immortalized human dermal microvascular endothelial cells (HMEC-1) for the study of HDL functionality

**DOI:** 10.1186/s12944-018-0695-7

**Published:** 2018-03-09

**Authors:** Mónica Muñoz-Vega, Felipe Massó, Araceli Páez, Elizabeth Carreón-Torres, Hector A. Cabrera-Fuentes, José Manuel Fragoso, Nonanzit Pérez-Hernández, Laurent O. Martinez, Souad Najib, Gilberto Vargas-Alarcón, Óscar Pérez-Méndez

**Affiliations:** 1 Molecular Biology Department, Instituto Nacional de Cardiología “Ignacio Chávez”, Juan Badiano 1, Sección XVI, 14080 Mexico City, Mexico; 2 Physiology Departments, Instituto Nacional de Cardiología “Ignacio Chávez”, Mexico City, Mexico; 3 Cardiovascular and Metabolic Disorders Program, Duke-NUS Graduate Medical School, Singapore, Singapore; 4 Institute of Biochemistry, Medical School, Justus-Liebig-University, Giessen, Germany; 5 National Heart Centre Singapore, National Heart Research Institute Singapore, Singapore, Singapore; 6 Institute of Metabolic and Cardiovascular Diseases, I2MC, Inserm, UMR, 1048 Toulouse, France

**Keywords:** HDL, HUVEC, Adhesion molecules, Inflammation, Endoglin, Atherosclerosis

## Abstract

**Background:**

Primary cultures endothelial cells have been used as models of endothelial related diseases such atherosclerosis. Biological behavior of primary cultures is donor-dependent and data could not be easily reproducible; endothelial cell lines are emerging options, particularly, human dermal microvascular endothelial cells (HMEC-1), that should be validated to substitute primary cultures for the study of HDL functions.

**Methods:**

Morphology, size and granularity of cells were assessed by phase contrast microscopy and flow cytometry of HMEC-1. The adhesion molecules, ICAM-1and VCAM-1 after TNF-α stimulation, and endothelial markers CD105 endoglin, as well as HDL receptor SR-BI were determined by flow cytometry. Internalization of HDL protein was demonstrated by confocal microscopy using HDL labeled with Alexa Fluor 488. HUVECs were used as reference to compared the characteristics with HMEC-1.

**Results:**

HMEC-1 and HUVEC had similar morphologies, size and granularity. HMEC-1 expressed endothelial markers as HUVECs, as well as functional SR-B1 receptor since the cell line was able to internalize HDL particles. HMEC-1 effectively increased ICAM-1 and VCAM-1 expression after TNF-α stimulation. HUVECs showed more sensibility to TNF-α stimulus but the range of ICAM-1 and VCAM-1 expression was less homogeneous than in HMEC-1, probably due to biological variation of the former. Finally, the expression of adhesion molecules in HMEC-1 was attenuated by co-incubation with HDL.

**Conclusion:**

HMEC-1 possess characteristics of endothelial cells, similar to HUVECs, being a cell line suitable to evaluate the functionality of HDL vis-à-vis the endothelium.

## Background

Endothelium has been focused as the site of initiation of atherosclerosis [1–6]; endothelial cells perform important inflammatory, apoptotic and thrombotic activities in order to maintain vascular homeostasis [7–10]. To elucidate the cellular and molecular mechanisms of pathologies related with the endothelium such as atherosclerosis, primary cultures of bovine aortic endothelial cells (BAECs) or human umbilical vein endothelial cells (HUVECs) have been used as models. However, the biological responses of endothelial cells to different stimulus are donor-dependent [11–15], thus the achievement of reproducible results becomes challenging. This is one of the major disadvantages of these primary cultures and stresses the validity of conclusions obtained with HUVEC of BAECs. In addition, endothelial cell conservation, isolation, as well as a nutritional requirements make these primary cultures technically demanding [15]. Besides the biological variability, and the economic and technical disadvantages, ethical considerations and legislations in some countries make difficult the donation of umbilical cords to isolate HUVECs. However, HUVECs are still considered the reference model in almost several endothelial-based studies.

Some endothelial cell lines have been developed as alternative to primary cell culture with advantages in life span and growth requirements [15]. An example of these alternatives are HMEC-1 cells, a microvasculature endothelial cell line developed from human foreskins and transformed with a vector designated as pSVT. This construct is based in PBR322 containing the sequences encoding the transforming protein SV40 large T, and its expression is driven by the Rous sarcoma virus long terminal repeat [16].

HMEC-1 has a life span 10 times longer than primary culture and their nutritional exigencies are lower. Additionally, HMEC-1 cell line retains endothelial phenotypical characteristics like expression of von Willebrand factor, uptake of acetylated-LDL, and expresses several endothelial markers and adhesion molecules [16]. These characteristics suggest that HMEC-1 would be a suitable model to study lipoproteins-endothelium interactions studies, specifically with lipoproteins, an approach that has not been explored yet. Therefore, in the present study we analyzed the feasibility of using HMEC-1 cell line as alternative for the study of some HDL properties *vis-à-vis* the endothelial cells, i.e. regulation of adhesion molecules and HDL internalization.

## Methods

### Reagents

Fetal calf serum were from GE heathcare (Logan, Utah) and Corning (New York, NY) L-glutamine, N-[2-hydroxyethylpiperazine-N_0_-[ethanesulfonic acid] (HEPES), endothelial cell growth supplement and porcine heparine were purchased from Sigma Chemical Co. (St. Louis,MO). M-199 medium with phenol red, MCDB-131 medium with phenol red, type II collagenase, liquid trypsin EDTA were from Gibco Laboratories (Grand Island, NY). Recombinant TNF-α was from Boehringer-Mannheim Bioquímica (Mexico City). APC conjugated anti-CD105, anti-VCAM-1 labeled with PE and anti-ICAM-1 associated with FITC were purchased from BioLegend (San Diego, CA) and anti- SR-B1 from Novus Biologicals (Littleton, CO). Goat anti-mouse IgG secondary antibody conjugated with PE from Santa Cruz Biotechnology (Dallas, TX). Protein labeling kit molecular probes Alexa 488 was purchased from Life technologies (Eugene, OR).

### Cell culture

HUVECs were isolated by treatment with 0.2% type II collagenase and cultured using M-199 medium with phenol red supplemented as previously described [12]. Briefly, HUVECs were cultured at 37 °C in a 7% CO_2_ humidified atmosphere, in medium M-199 with phenol red and 20% fetal calf serum, penicillin, streptomycin, L-glutamine 10 mM, hydrocortisone 1 μg/mL, endothelial cell growth supplement (40 μg/mL) and heparin. The experiments were performed using pools composed of three different umbilical cords from healthy donators without personal and familiar history of cardiovascular diseases.

HMEC-1 (ATCC CRL-3243) were cultured at 37 °C in a 7% CO_2_ humidified atmosphere using MCDB-131 medium with phenol red and supplemented with 15% fetal calf serum, penicillin, streptomycin, L-glutamine 10 mM, hydrocortisone 1 μg/mL, endothelial cell growth supplement (20 μg/mL).

Morphology and granularity were assessed using cells without markers or stimuli using flow cytometry in a BD FACS Calibur equipment (Singapore).

### Expression of adhesion molecules and endothelial markers

To induce the expression of vascular cell adhesion molecule-1 (VCAM-1) and intercellular adhesion molecule-1 (ICAM-1), cells were recovered using PBS solution using 0.5% trypsin/EDTA. After incubation, medium was changed by MCDB-131 or M-199 supplemented with 7% lipid poor serum prepared by ultracentrifugation (starvation medium) [17]. Then, cells were incubated during 5 h with TNF-α at different concentrations.

After treatment with TNF-α, cells were recovered using collagenase, washed and suspended. Cells were fixed with 3.7% paraformaldehyde in PBS and then labeled by incubation for 1 h with fluorophore-conjugated anti-ICAM-1, anti-VCAM-1, anti-CD105 antibodies. Alternatively, anti-scavenger receptor class B member 1 (SR-B1) and the corresponding secondary anti-mouse phycoerythrin (PE) conjugated antibody were used to determine this receptor. Antibodies were washed and then cells were analyzed by flow cytometry.

### HDL isolation and labeling

We obtained plasma of 81 voluntary healthy donors from “Instituto Nacional de Cardiologia Ignacio Chávez” who agreed to participate in our study trough signing the correspondent informed consent approved by the institutional research committee. Subjects were excluded if they had personal history of diabetes, hypertension, chronic kidney disease, liver disease, anemia, thyroid abnormalities, if they were taking any medication or if they present any dyslipidemia. Samples were divided by day of obtaining in 7 pools each with an average of 11 samples. HDL were isolated by sequential ultracentrifugation as reported before [18]. Cell stimulation with HDL was performed using a final concentration of 40 mg/dL of cholesterol for each condition.

### HDL internalization assay

HDL protein was labeled using Alexa 488 Molecular Probes according to the specifications of manufacturer (Life Technologies, Eugene, OR). HDL internalization assays were performed as described before [19] with slight modifications. Briefly, cells were starved and incubated in all steps of the assay with medium MCDB or M-199, accordingly with cell type, containing 7% of lipid poor fetal calf serum, cells were washed with PBS and images were obtained by confocal microscopy using a LSM-700 Zeiss equipment (Baden-Württemberg).

### Statistical analysis

Results were expressed as mean fluorescence intensity obtained after analysis of 5000 events. Comparison between groups was performed using Kruskall-Wallis non-parametric test using Graph Pad Prism 5.0 software.

## Results

**Fig. 1:**
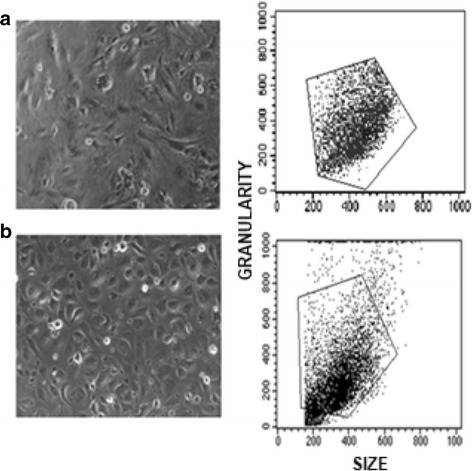
Characteristics of HMEC-1 (**a**) compared with HUVEC (**b**). Morphology was assessed with phase contrast microscopy (left). Size and granularity of cell cultures was determined by flow cytomety (right). Representative images from phase-contrast microscope at 100×. The cytometer setting parameters were the same for both types of cells

**Fig. 2:**
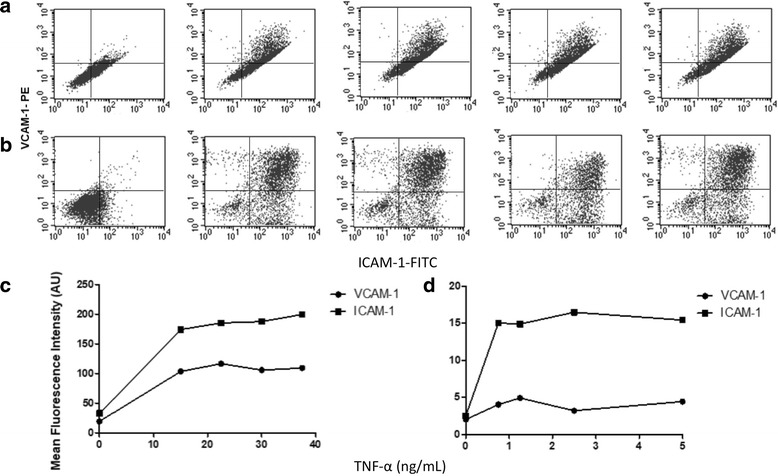
Induction of ICAM-1 and VCAM-1 in HMEC-1 (**a**) and HUVECs (**b**) by increasing concentrations of TNF-α. ICAM-1 and VCAM-1 presence in cell membranes was determined by flow cytometry; ICAM-1 and VCAM-1 antibodies were labeled with FITC and PE, respectively. Lower panel, dose-response curves of HMEC-1 (**c**) and HUVECs (**d**)

Morphology, size and granularity were similar for both, HMEC-1 and HUVECs (Fig. 1). We determined the optimal concentration of TNF-α to induce ICAM-1 and VCAM-1, by a dose-response curve (Fig. 2); we measured the response trough dot plots in terms of quantity of both, ICAM-1 and VCAM-1 expressed as double positive cells (right up quadrant) for HMEC-1 (Fig. 2A) and HUVEC (Fig. 2B). The optimal response of HMEC-1 to TNF-α was reached with a concentration of 15 ng/mL (Fig. 2C); higher doses of TNF-α did not induce a greater expression of adhesion molecules. Therefore we used the concentration of 15 ng/mL of TNF-α in further experiments.

Concerning HUVECs, the dot plots showed a wider range of TNF-α-induced expression of ICAM-1 and VCAM-1 (Fig. 2B); responses were observed from doses of 0.75 ng/mL of TNF-α but the dose-response effect was not as regular as for HMEC-1. We used 0.75 ng/mL of TNF-α concentration for the subsequent experiments with HUVECs.

**Fig. 3:**
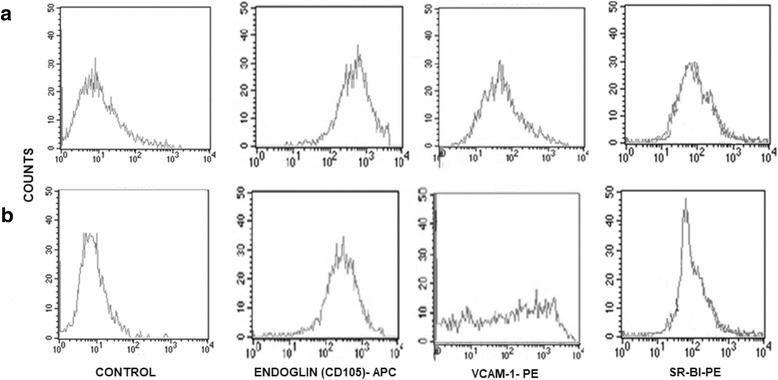
Co-expression of endothelial markers and SR-B1. Membrane levels of endoglin (CD-105), VCAM-1 and SR-B1 were measured by flow cytometry in (**a**) HMEC-1 and (**b**) HUVEC after we treated them with TNF-α at final concentrations of 15 and 0.75 ng/mL respectively. Endoglin (CD-105) labeled with APC, VCAM-1 and SR-BI labeled with PE

We further search for the expression of VCAM-1 and CD105, also named endoglin, characteristic of endothelial cells. We observed a co-expression of VCAM-1 and endoglin in both types of endothelial cells after TNF-α stimulation. Endoglin was expressed in the same extent in both types of cells and histograms were very similar for a constitutive marker (Fig. 3). In contrast, VCAM-1 was expressed in a broad range in HUVEC pool of 3 healthy donors whereas such expression was more homogeneous in HMEC-1 cells (Fig. 3).

**Fig. 4:**
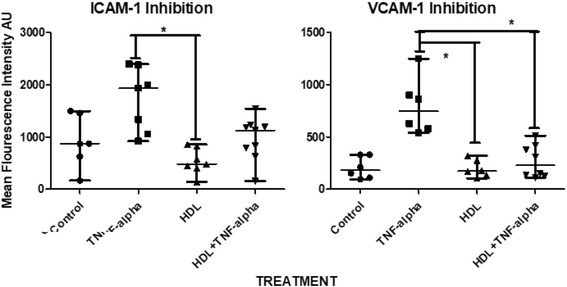
ICAM-1 and VCAM-1 inhibition by HDL in HMEC-1. Measures were performed by flow cytometry using a FITC-conjugated ICAM-1 antibody and a PE VCAM-1 associated antibody. *n* = 7 pools by condition, *P < 0.005 Kruskall-Wallis. Bars represent median and range of data

We further quantified membrane SR-B1 (Fig. 3); both, HUVEC and HMEC-1 were positive for this receptor at similar levels of expression (Fig. 3). To explore functional aspects of HDL on endothelium function, we incubated HMEC-1 with 7 different HDL pools, TNF-α, or both. ICAM-1 and VCAM-1 tended to be expressed below the basal levels (constitutive expression) when cells were co-incubated with HDL, but the differences did not reach statistical significance. In contrast, HDL significantly attenuated the expression of TNF-α-induced VCAM-1, whereas ICAM-1 HDL inhibition did not reach statistical significance (Fig. 4).

**Fig. 5:**
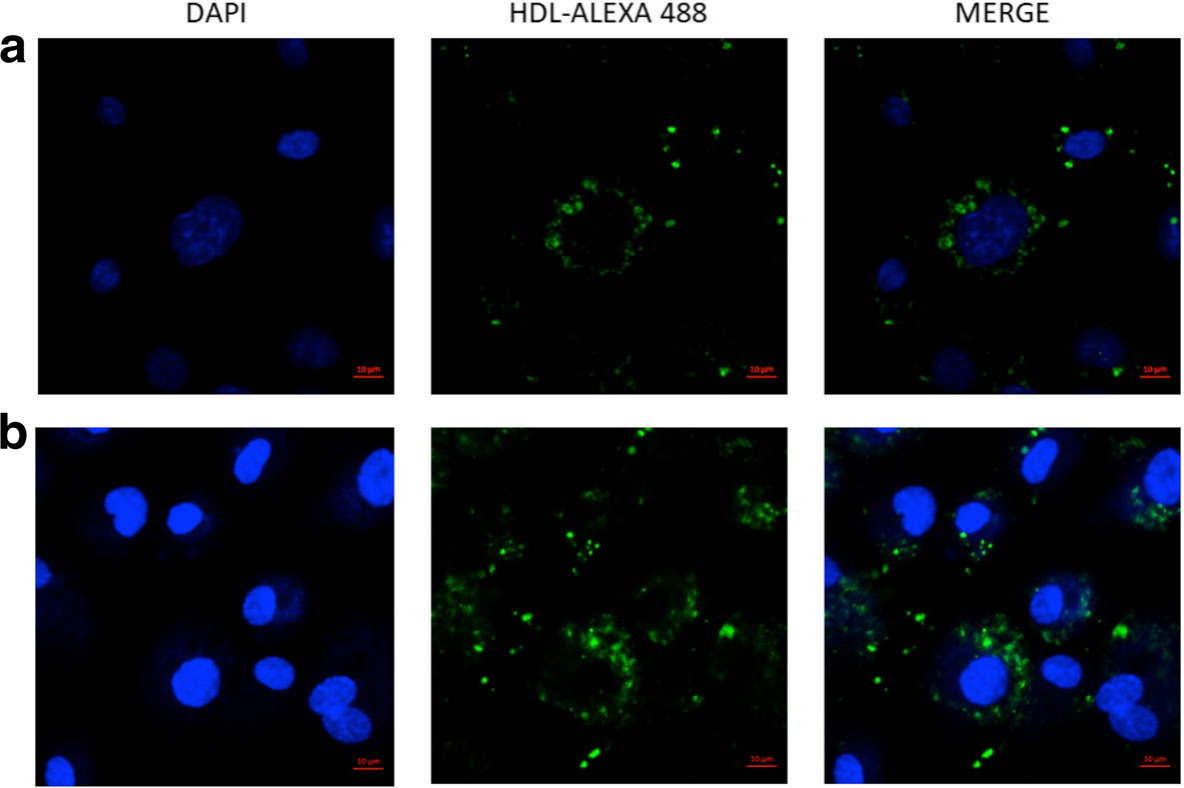
Confocal microscopy showing HDL internalization in HMEC1 (**a**) and HUVECs (**b**). Representative image of experiments using HDL labeled in the protein moiety with Alexa 488 (green) and nuclei stained with DAPI (blue)

SR-B1 expression in HMEC-1 is relevant in terms of HDL endothelial functionality; previous studies [20, 21] using primary cultures demonstrated that HDL are internalized by endothelial cells. Therefore, we performed HDL internalization assays in HMEC-1 cells and HUVECs using HDL-protein labeled with Alexa Flour 488. Confocal microscopy showed that HMEC-1 and HUVEC are able to internalize HDL (Fig. 5). Interestingly, both endothelial models showed that HDL protein was located in discrete granules inside the cytoplasm.

## Discussion

In this study we demonstrated that HMEC1 possess similar properties than HUVECs vis-à-vis HDL interaction. HUVECs have been extensively used for the study of lipoprotein properties with regard to endothelial cells. However, the use of HUVECs represents some disadvantages, particularly related with reproducibility, due to the inter-individual biological variability [11–14] and technical complexity. For this, in the present work we propose the use of HMEC-1 a cell line of endothelium as alternative of HUVECs.

We first demonstrated that both cell types have similar size and granularity; size and granularity are suitable parameters to identify cell types. Granularity is a measure of cell complexity and depends of nucleus size and the presence of cytoplasmic organelles and vesicles. Therefore, the structure and complexity of HMEC-1 and HUVECs are comparable. Interestingly, HMEC-1 were more homogeneous than HUVECs in terms of size and granularity, suggesting less variability of experimental data obtained with these cells.

ICAM-1 and VCAM-1 are cell adhesion molecules expressed by the endothelium with important roles in cell migration during inflammation. ICAM-1 is expressed constitutively and strongly induced by stimulus like TNF-α, whereas VCAM-1 is mainly expressed after the pro-inflammatory stimulus [22, 23]. In this context, HMEC-1 reached a maximum expression of ICAM-1 and VCAM-1 with 15 ng/mL TNF-α, and such expression remained stable with higher concentrations of TNF-α. In contrast, HUVECs showed a variable expression at increasing doses of the stimulus. The inter-individual variability of HUVECs may be the cause of the less homogeneous dose-response of HUVECs in these experiments, even if we use a pool of umbilical cords from three different donors to obtain more representative results than those obtained with single donor samples. These observations support the idea that HMEC-1 cultures are helpful to obtain more reproducible results. However, it should be emphasized that the HMEC-1 are less sensitive to stimulus, the amount of TNF-α to reach a maximum response in was about 20 times the concentration required for HUVECs. These results should be considered when using HMEC-1 to evaluate endothelial response to inflammatory stimulus.

One of the aims of this study is to determine whether HMEC-1 are suitable for evaluating some HDL properties with regard to endothelial cells; to the best of our knowledge, there are not previous reports with this purpose. Interaction of HDL with cells is often mediated by SR-B1 also named CLA-1. This is the main known receptor for HDL expressed by the liver, steroidogenic tissues and recently, it has been reported in endothelium [24, 25]. For this reason, we first look for the expression of such receptor in HUVEC and HMEC-1; our results clearly showed that both type of cells expressed SR-B1 in a similar extent, supporting again the idea that HMEC-1 are useful for the study of HDL properties. In addition, the endothelial marker CD105, also known as endoglin, was expressed on the membrane of both types of endothelial cells. CD105 is a transforming growth factor-beta (TGF-beta) co-receptor expressed mainly on endothelial cells and involved in cardiovascular development, angiogenesis, and vascular remodeling [26].

Once we demonstrated that HMEC-1 express key markers of endothelium and the HDL receptor, SR-B1, we further analyzed the usefulness of this cell line to evaluate the anti-inflammatory property of HDL related with the expression of adhesion molecules induced by TNF-α, [27, 28]. Previous studies have demonstrated that this property of HDL is impaired in some individuals and may be associated with increased risk of coronary heart disease [29–31]. We performed these experiments using pools of plasma obtained from at least 12 different donors in order reduce the heterogeneity of the samples in terms of the regulation of adhesion molecules. We observed that HDL clearly inhibited VCAM-1 expression when incubated with TNF-α as expected, whereas ICAM-1 only showed a tendency to a lower expression. Interestingly, the incubation of HDL inhibited expression of adhesion molecules below control levels. This experiment demonstrated HMEC-1 are suitable for HDL anti-inflammatory function studies as well.

A potential mechanism involved in the regulation of endothelial cell function by HDL, may be the internalization of these lipoproteins as previously demonstrated in HUVECs and bovine aortic endothelial cells [21, 32–34]. Therefore, we look for the capacity of HMEC-1 to internalize HDL particles by labeling the protein moiety; our data clearly showed that HMEC-1 were able to internalize HDL particles. Interestingly, HDL is likely to be inside vesicles in perinuclear area, similar to previous reports [20, 21]. These previous studies have demonstrated that HDL vesicles did not present any typical marker of organelles from secretory pathway, suggesting an additional mechanism for HDL; nevertheless, internalization process at the moment is not totally understood [35] and requires further investigation.

## Conclusion

In this study we demonstrated that HMEC-1 possess characteristics of endothelial cells, in some cases more homogeneous than HUVECs, supporting the idea that this cell line is suitable to evaluate the functionality of HDL vis-à-vis the endothelium.

## Abbreviations

BAECs: bovine aortic endothelial cells
CD105: cluster of differentiation 105
HDL: high-density lipoproteins
HMEC-1: human dermal microvascular endothelial cells-1
HUVECs: human umbilical vein endothelial cells
ICAM-1: intercellular adhesion molecule-1
SR-B1: scavenger receptor class b type 1
TNF-α: transforming necrosis factor alpha
VCAM-1: vascular cell adhesion molecule-1
